# Evaluation of the system accuracy of frameless stereotactic radiosurgery using a combination of cone beam CT, six degrees of freedom couch, and surface image‐guided systems

**DOI:** 10.1002/acm2.70082

**Published:** 2025-03-22

**Authors:** Hao‐Wen Cheng, Jonathan Li, Sheng‐Hsuan Sun, Guanghua Yan, Chihray Liu

**Affiliations:** ^1^ Department of Radiation Oncology College of Medicine University of Florida Gainesville Florida USA

**Keywords:** optical surface imaging system, quality assurance, stereotactic radiosurgery, surface‐guided radiation therapy

## Abstract

**Purpose:**

This study aims to evaluate the accuracy of the frameless linear accelerator‐based stereotactic radiosurgery (FSRS) system incorporating cone‐beam CT (CBCT), six degrees of freedom (6‐DoF) couch, room laser, and surface image‐guided (SG) systems. It focuses on assessing the FSRS system's accuracy and ability to detect position errors using head phantoms at different couch angles. Turntables were used to simulate the couch rotation to overcome the limitation of the available couch rotation angles for a 360° CBCT scan.

**Methods:**

Two head phantoms, each positioned on its respective turntable, were used for measurements, with the turntables placed on the couch. Factors affecting the evaluations of the FSRS system's accuracy were analyzed, including quality assurance (QA) procedures for the SG system, the automatic CBCT‐CT registration method, the CBCT clip box volume, and the selected region of interest (ROI) size in the SG system. Discrepancies in isocenter shifts between CBCT and SG systems were measured to evaluate the FSRS system's accuracy and its ability to correct isocenter shifts at different turntable angles. The FSRS system's ability to detect position errors at different turntable angles was also evaluated by introducing ± 2.0 mm translational errors.

**Results:**

With the appropriate CBCT‐CT registration method and ROI size of the SG system, the accuracy evaluations of the FSRS system indicated average discrepancies between the readouts from the CBCT and the SG system ≤ 0.9 mm/0.8° for the head phantoms. Error simulation demonstrated that the FSRS system was able to detect position errors when 2 mm translational errors were intentionally introduced, with most average discrepancies < 1 mm/1°.

**Conclusion:**

This study introduces an innovative approach to quantifying the impact of couch rotation on the FSRS system using head phantoms with turntables. The overall accuracy of the FSRS system was on the order of 1.1 mm/1°.

## 1 INTRODUCTION

Stereotactic radiosurgery (SRS) involves delivering a concentrated high radiation dose over a limited number of fractions (typically < 5). Due to the high target dose and the necessity to spare normal tissues, accurate and precise dose delivery is crucial in SRS. Conventional SRS, such as Gamma Knife and floor stand linear accelerator (linac)‐based SRS, uses a rigid head frame to immobilize the patient's head, ensuring high mechanical accuracy in tumor localization. Modern frameless linear accelerator (linac)‐based SRS (FSRS) systems, which integrate image‐guided devices such as orthogonal kV x‐rays and cone‐beam computed tomography (CBCT) along with an immobilization mask, not only offer high‐precision and high‐accuracy treatment but also enhance patient comfort during treatment.^1^, ^2^, ^3^, ^4^, ^5^ Furthermore, the use of six degrees of freedom (6‐DoF) couch system can improve patient positioning accuracy in FSRS treatment.^6^, ^7^, ^8^

In recent years, a surface‐guided radiation therapy (SGRT) technique with an open‐face immobilization mask has been introduced for FSRS.^9^, ^10^, ^11^, ^12^, ^13^ This technique relies on an optical surface imaging system to continuously monitor the surface of a patient. Therefore, it can provide real‐time patient position during patient setup, and patient position monitoring during coplanar and noncoplanar FSRS treatment.

The American Association of Physicists in Medicine task group 302 (TG‐302) report notes that during FSRS treatment, when the treatment couch is rotated away from 0°, there is a potential for the SG camera units to experience reduced visibility of a portion of the region of interest (ROI), especially at the couch angles of ± 90°, which could result in decreased accuracy in patient position monitoring for noncoplanar FSRS treatment.^14^ Yock et al. demonstrated that for the single isocenter per target (SIPT) FSRS plans, there was a median reduction of 2.3% in target coverage with 2.0 mm/2.0° positioning errors, while the target coverage remained nearly unchanged with 1.0 mm/1.0° positioning errors. However, for the single‐isocenter multiple‐target (SIMT) FSRS plans, the median target coverage decreased by 39.8% with 2.0 mm/2.0° positioning errors and by 0.5% with 1.0 mm/1.0° positioning errors.^15^ The TG‐302 report also suggests that tighter tolerances of patient positioning (≤ 1 mm/0.5°) should be adopted for the SIMT FSRS technique to mitigate the dosimetric impact caused by patient positioning errors.^14^ Due to the high demand for accuracy and precision in FSRS, it is essential to ensure that patient positioning accuracy remains within acceptable tolerance limits throughout treatment, particularly for noncoplanar FSRS treatment.

For FSRS treatment, CBCT is typically the clinical practice standard for final patient setup.^14^, ^16^, ^17^ Several studies have evaluated the accuracy of the SG system for FSRS treatment using a head phantom compared to kV imaging systems at different couch angles, but only at limited couch angles when compared to CBCT.^18^, ^19^, ^20^

In our clinical practice, our FSRS system incorporates different components: a linac equipped with CBCT and MV beam, a 6‐DoF couch, room lasers, and an SG system. This study aims to evaluate the accuracy of the FSRS system by comparing isocenter shifts and the ability to detect position errors between the CBCT and SG systems at different couch angles using two head phantoms. It investigates whether the SG system alone is sufficient for FSRS treatment. However, performing a 360°CBCT scan is limited by the available couch rotation angles (typically within ± 20°) due to collision concerns. To overcome this limitation, turntables placed on the 6‐DoF couch were used to simulate couch rotation, allowing us to perform 360°CBCT scans at any turntable angle. Furthermore, we investigated different potential factors that may affect the evaluation of the FSRS system's accuracy, including the quality assurance (QA) procedure for the SG system, the CBCT‐CT image registration method, the CBCT clip box volume, and the selected ROI size in the SG system. Our primary focus in this study was to quantify the impact of couch rotation on the accuracy of the FSRS system. Other potential impacts, such as the camera occlusion from the gantry or other devices, delineation errors, and intrafraction motion, are beyond the scope of this study. The dosimetry accuracy is also outside the scope of this study.

## 2 MATERIALS AND METHODS

### 2.1 System description

In this study, a Versa HD linac equipped with the HexaPOD Evo RT (HexaPOD) system (Elekta, Stockholm, Sweden) and an SG system, Catalyst^+^ HD (C^+^HD) (C‐RAD, Uppsala, Sweden) were used.

The HexaPOD system, a 6‐DoF robotic patient positioning system, consists of an iGUIDE tracking system featuring an infrared camera, an iGUIDE reference frame with infrared‐reflective markers, and a HexaPOD robotic couch. The iGUIDE tracking system can monitor the position of the iGUIDE reference frame installed on the robotic couch during patient positioning, ensuring accurate patient positioning in 6‐DoF at submillimeter levels.

The C^+^HD, a 3D optical surface imaging system, consists of three ceiling‐mounted camera units at fixed angles, enabling continuous surface detection of the patient. During patient setup, the C^+^HD assists in patient positioning by calculating posture and position errors and then projecting a distance color map onto the patient's surface. This map provides guidance on which specific body parts need adjustment. During treatment, the C^+^HD can monitor the patient's position and calculate the isocenter shift in 6‐DoF.

All the CBCT scans were performed using the kV‐CBCT x‐ray volume imaging (XVI) system integrated into the Versa HD linac. For these scans, full gantry rotation (360°) was used with a small field of view (S20) and F0 filters.

### 2.2 System integration

A series of QA procedures was implemented to ensure the alignment of the isocenters among the different systems within acceptable tolerance limits, including the Versa HD linac (XVI CBCT and MV beam), the HexaPOD, the room laser, and the C^+^HD systems.

#### 2.2.1 MV‐kV coincidence QA

This QA procedure consists of two steps:

1. Couch movement accuracy check:

In the first step, the couch with the ball bearing (BB) phantom placed at its end was moved to a predetermined position (1.5 cm from the isocenter in each direction). Subsequently, the first CBCT images of the BB phantom were acquired for manual registration with the reference CT image (CBCT‐CT image registration). Translational shifts were applied to the couch based on the registration result. A second CBCT scan of the BB phantom was then performed, and the second set of couch shift values was obtained through the second manual CBCT‐CT image registration. The couch movement accuracy was evaluated based on these second couch shift values, and these values in the translational directions should be within the tolerance limit of 0.5 mm.

2. MV‐kV coincidence check:

In the second step, the MV isocenter was verified using the BB phantom with 6 MV photon beams (10 × 10 cm^2^ field size [FS]) and the electronic portal imaging device (EPID). Eight EPID images were acquired at gantry angles of 0°, 90°, 180°, and 270° with collimator angles set to ± 90°. An in‐house MATLAB (MathWorks, Natick, MA, USA) program analyzed the EPID images and calculated the translational discrepancies between the BB center and the MV isocenter. Then, the MV‐kV coincidence (vector difference) can be calculated based on the second couch shift values and the translational discrepancies between the BB center and the MV isocenter. The MV‐kV coincidence should be within 0.5 mm. After the MV‐kV coincidence check, the CBCT isocenter can be regarded as the standard for verifying the isocenters of the other systems.

In addition, we also conducted the Winston–Lutz (WL) test using the Lucy 3D phantom (Standard Imaging, Inc., Middleton, WI, USA) with a 19‐mm SRS cone and a 2 × 2 cm^2^ multi‐leaf collimator (MLC)‐shaped FS. The gantry and collimator settings were the same as mentioned above. The WL test was repeated at least seven times for both the cone and the MLC‐shaped FS.

#### 2.2.2 QA for coincidence of HexaPOD, XVI CBCT, and room laser systems

The alignment check of the room laser system with the CBCT system was initiated as the first step. The MIMI phantom (Standard Imaging, Middleton, WI, USA) was set up using the room lasers. Subsequently, a CBCT scan of the MIMI phantom was performed. To realign the center of the MIMI phantom to the CBCT isocenter, 6‐DoF HexaPOD couch shifts were applied based on the automatic CBCT‐CT image registration result.

After implementing the HexaPOD couch shifts, the alignment between the CBCT isocenter and the room laser isocenter (i.e., MIMI phantom center vs. room laser isocenter) was checked. The room lasers were adjusted to align with the MIMI phantom's crosshairs. Once the room laser isocenter adjustments were complete, we mounted the iGUIDE reference frame on the HexaPOD couch. The alignment between the iGUIDE reference frame and the room lasers was verified, and the HexaPOD couch was adjusted as needed to align the crosshairs of the iGUIDE reference frame to the room lasers.

These procedures ensure that the room laser isocenter matches the CBCT isocenter and that the HexaPOD system isocenter matches the room laser isocenter within the tolerance limits of 0.5 mm.

#### 2.2.3 C^+^HD routine QA

The C+HD routine QA ensures the alignment between the C+HD isocenter and the CBCT isocenter, and it consists of two steps:

1. SG camera unit drift check:

In the first step, we aligned the marks on the Daily Check device of model HZ‐023 (C‐RAD, Uppsala, Sweden) to the room lasers. Then, the C^+^HD system would check for any drift in the SG camera units that may have occurred since the previous daily check or routine QA in 6‐DoF.

2. Alignment between the C^+^HD and the radiation isocenters using a verification imaging system (MV, CBCT, or kV):

The CBCT is used as the verification imaging system in our clinical practice. In the second step, we aligned the QUASAR Penta‐Guide (PG) phantom (Modus Medical Devices Inc., London, Canada) to the CBCT isocenter. This alignment was achieved by applying couch shifts based on the result of automatic CBCT‐CT image registration from a CBCT scan. The C^+^HD system then aligned its isocenter with the CBCT isocenter, considering the deviation between these two isocenters and the drift results obtained in the first step.

#### 2.2.4 HexaPOD couch level verification

**FIGURE 1:**
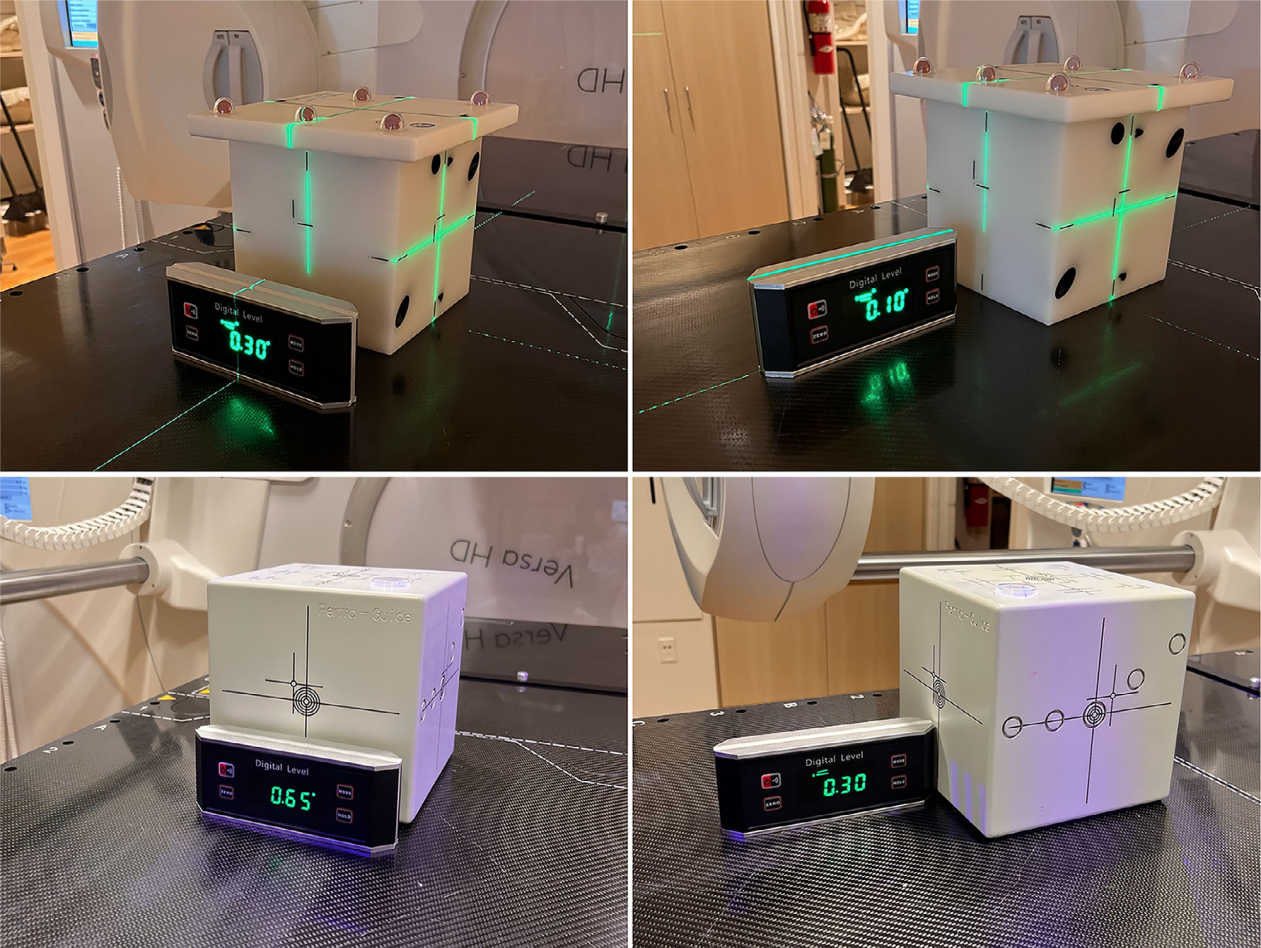
Digital level for verifying HexaPOD couch level after alignment checks with MIMI phantom and Penta‐Guide phantom.

For the QA procedures, which included the HexaPOD couch rotation correction, we placed a digital level near the phantom's position to verify the level of the HexaPOD couch after applying the HexaPOD couch shifts. This verification was performed after the previously mentioned alignment checks with the MIMI phantom and the PG phantom (in Sections 2.2.2 and 2.2.3), as shown in Figure 1. This procedure was repeated three times. The digital level was calibrated using a precision frame spirit level with a 0.3 mm/m accuracy (HAHN+KOLB Werkzeuge GmbH, Ludwigsburg, Germany), and the difference between the readouts was within 0.1°.

### 2.3 Head phantoms and surface reference image capture

**FIGURE 2:**
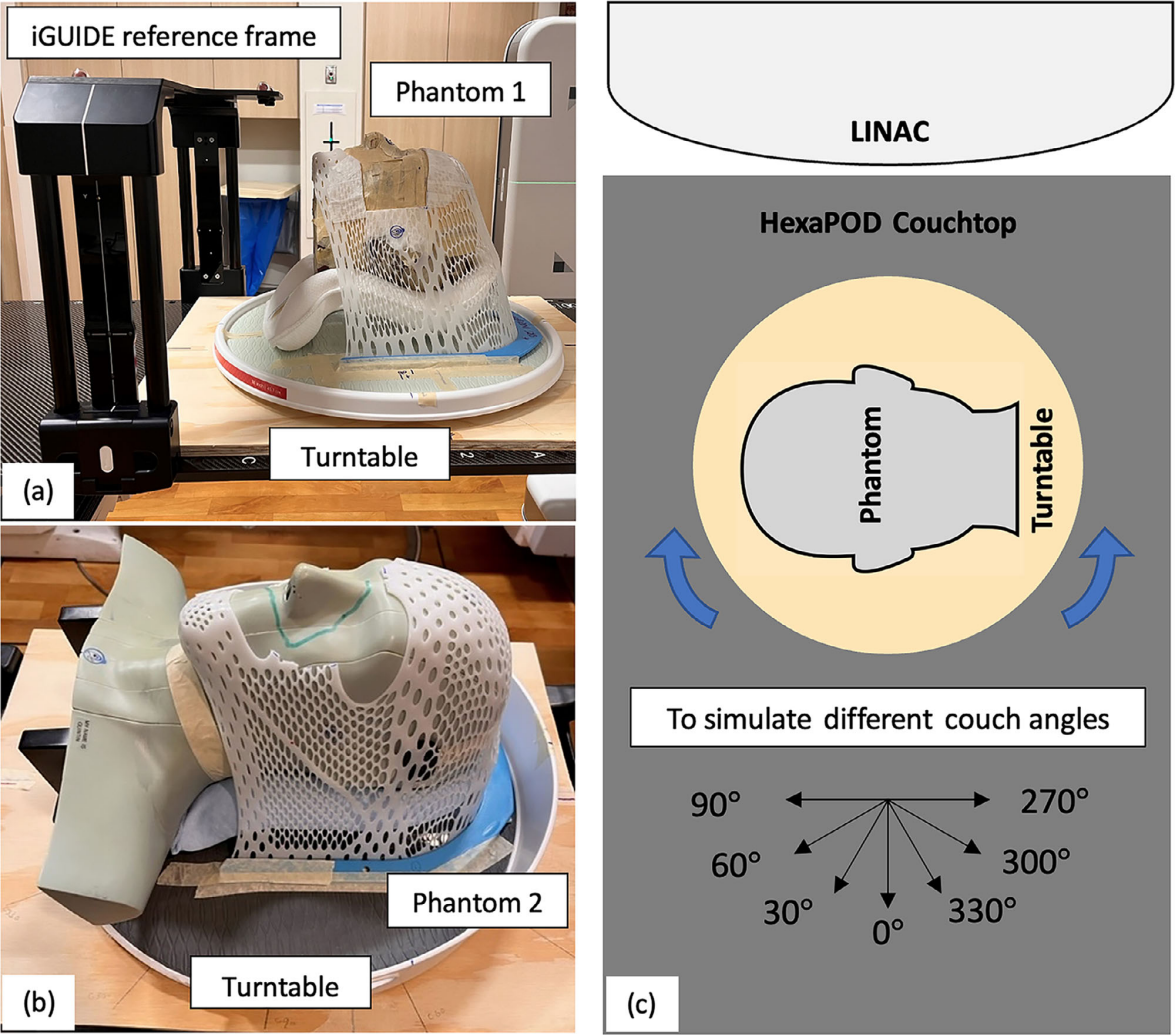
(a) Setup of RANDO head phantom (Phantom 1) for measurements. (b) Setup of modified Rando head‐and‐neck phantom (Phantom 2). (c) Illustration of the turntable with a phantom used to simulate the couch rotation.

This study used two head phantoms: a RANDO head phantom (Phantom 1, The Phantom Laboratory, Salem, NY, USA) and a modified Rando head‐and‐neck phantom (Phantom 2). Each phantom was placed on its respective turntable with a headrest and an open‐face immobilization mask, as shown in Figure 2. To reduce the turntable rotation offset to less than 2 mm, the treatment isocenter of each head phantom was aligned with the isocenter of each turntable in both the right–left and superior–inferior directions. These turntables can be rotated to a specific angle based on the C^+^HD rotation readout to simulate the couch rotation, allowing us to perform 360° CBCT scans at any turntable angle (Figure 2).

A Philips Brilliance Big Bore CT scanner (Philips Medical Systems, Madison, WI, USA) was used for the head phantom scans with a 1‐mm slice thickness. The Pinnacle Treatment Planning System (Philips, Version 16.4, Fitchburg, WI, USA) was used to generate reference images for the CBCT‐CT image registration.

In the C^+^HD system, the SRS treatment type was selected for both head phantoms. The head phantom on the turntable was placed on the HexaPOD couch and then positioned using CBCT at the couch angle of 0°. After the CBCT‐CT image registration, the 6‐DoF HexaPOD couch shifts were applied. A surface reference image was then captured using the C^+^HD.

### 2.4 Coordinate systems of CBCT and C^+^HD

The rotational directions of the CBCT coordinate system for rotation and pitch are opposite to those of the C^+^HD treatment coordinate system, while their translational directions are the same. We used the following equations to convert the C^+^HD treatment coordinate system to the CBCT coordinate system in the lateral (Lat.), longitudinal (Long.), and vertical (Vert.) directions:

(1) $$\mathrm{couch}\;\mathrm{angl}\;e^{\prime }=2\pi \times \mathrm{couch}\;\mathrm{angle}÷360$$

(2) $$\begin{array}{cc} \mathrm{Lat}. & =\cos\;\left(\mathrm{couch}\;{\mathrm{angle}}^{\prime }\right)\times \left(\mathrm{Lat}.\right)\\ & \;+\sin\;\left(\mathrm{couch}\;{\mathrm{angle}}^{\prime }\right)\;\times \left(\mathrm{Long}.\right) \end{array}$$

(3) $$\begin{array}{cc} \mathrm{Long}. & =-\sin\left(\mathrm{couch}\;{\mathrm{angle}}^{\prime }\right)\times \left(\mathrm{Lat}.\right)\\ & \;+\cos\left(\mathrm{couch}\;{\mathrm{angle}}^{\prime }\right)\;\times \left(\mathrm{Long}.\right) \end{array}$$

(4) Vert.=Vert.

### 2.5 CBCT‐CT image registration method

The automatic CBCT‐CT image registration method may affect the evaluation of the accuracy of the FSRS system. In this study, two automatic registration methods with a predefined clip box (where the registration occurs only within the clip box) were evaluated to determine the suitable registration method for head phantom images: Bone (T+R) mode (BM) using the chamfer matching algorithm^21^ for bone matching, and Gray value (T+R) mode (GM) using the gray level correlation ratio algorithm for voxel grayscale intensity value matching.^22^ CBCT scans were performed on the two head phantoms at seven different turntable angles (0°, 30°, 60°, 90°, 270°, 300°, and 330°). The discrepancies in isocenter shifts between the two different registration methods were then calculated. This procedure was repeated six times.

### 2.6 Accuracy of FSRS system based on two different routine QA procedures

According to the routine QA procedure in the C^+^HD system manual, the difference between the CBCT and the C^+^HD isocenters is adjusted by performing CBCT scans of the PG phantom. However, the room laser isocenter was defined as part of the QA/calibration procedure for the HexaPOD system in Section 2.2.2.

**FIGURE 3:**
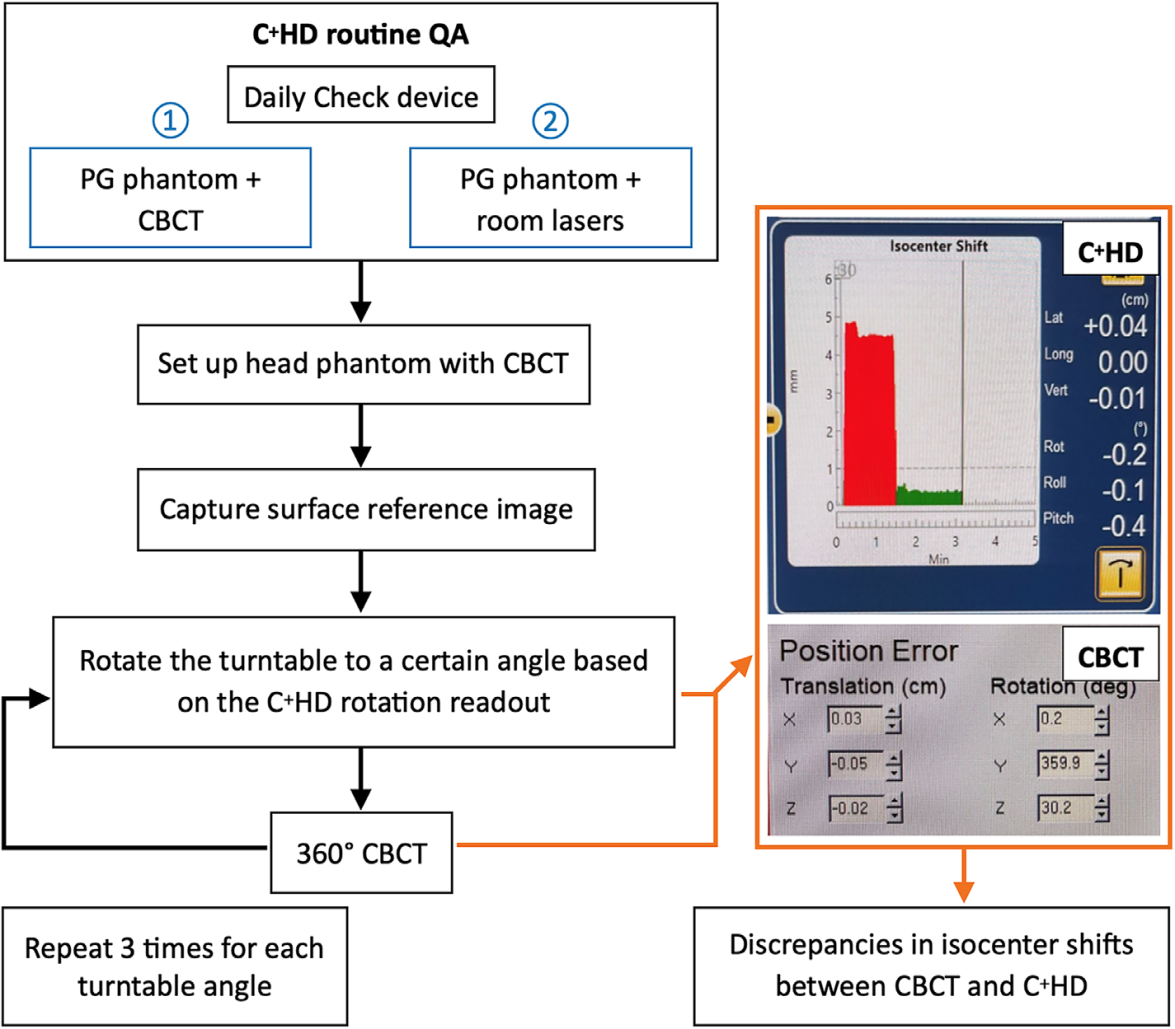
Flowchart of evaluating the accuracy of the FSRS system based on two different routine QA procedures. FSRS, frameless linear accelerator‐based stereotactic radiosurgery; QA, quality assurance.

To evaluate the accuracy of the FSRS system based on the above two different routine QA procedures, the first using the CBCT and the second using the room lasers for the PG phantom alignment, we measured the isocenter shifts from the CBCT and the C^+^HD at seven different turntable angles (0°, 30°, 60°, 90°, 270°, 300°, and 330°) using Phantom 1. The discrepancies in isocenter shifts between them were then calculated. This procedure was repeated three times. The workflow of this procedure is illustrated in Figure 3.

### 2.7 Accuracy of FSRS system

We used two methods to evaluate the accuracy of the FSRS system. In the first method, we measured the isocenter shifts from the CBCT and the C^+^HD using Phantom 1 and 2 at seven different turntable angles (0°, 30°, 60°, 90°, 270°, 300°, and 330°). We then calculated the discrepancies in isocenter shifts between them.

**FIGURE 4:**
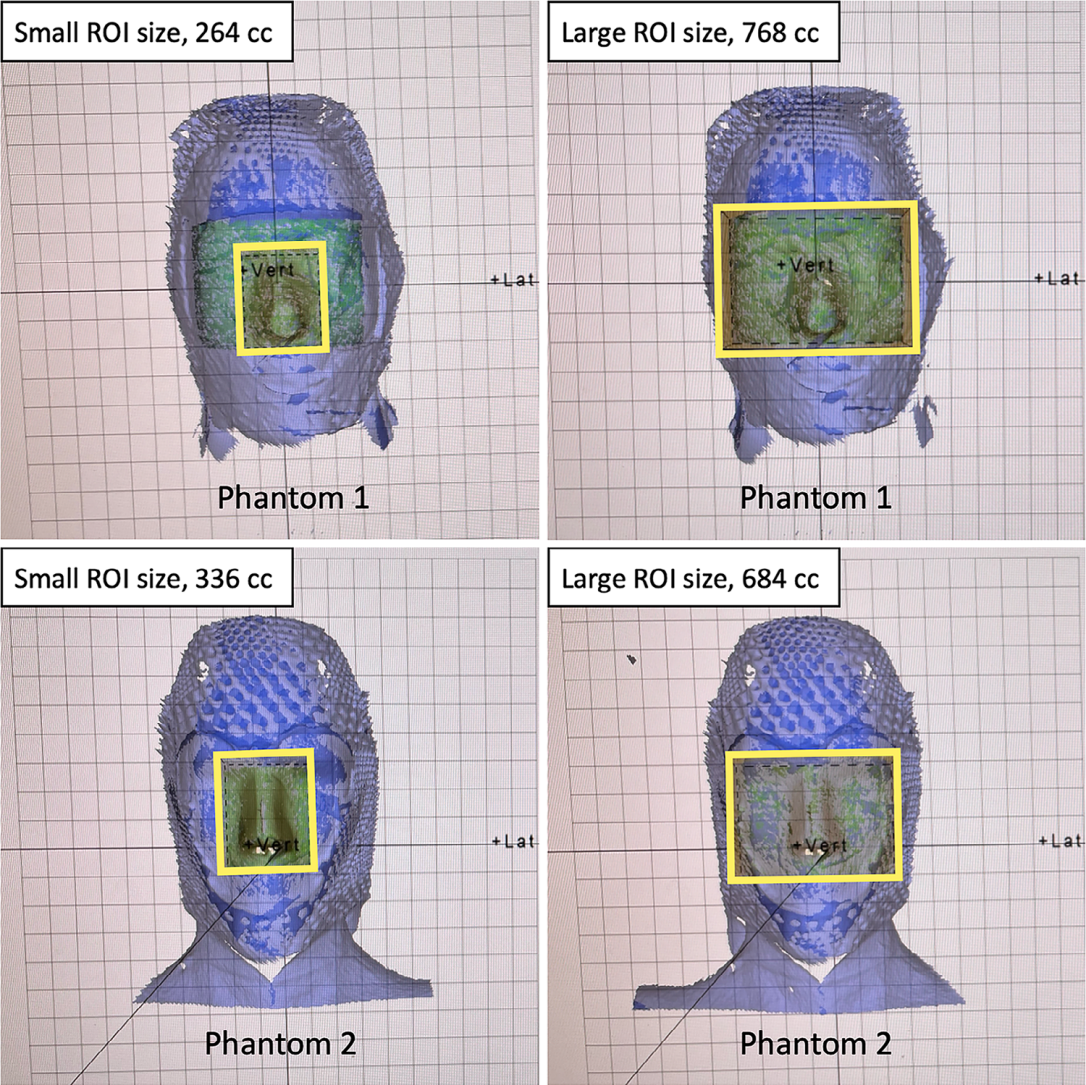
Two head phantoms with different ROI sizes; the yellow square represents the ROI. ROI. region of interest.

The size of the ROI selection in the C^+^HD system may affect the evaluation of the accuracy of the FSRS system.^14^ Therefore, we also evaluated the effect of ROI size using two different ROI sizes, a small one and a large one, as shown in Figure 4. The small ROI size encompassed the nose plus 1 cm margins in both lateral and longitudinal directions. This choice was made assuming that nose movement would be smaller than other facial parts (such as the eyes, mouth, etc.) during treatment, which can serve as a suitable surrogate for intra‐fractional motion detection. The large ROI size encompassed the eyes, nose, and cheeks. This procedure was repeated three times for both head phantoms.

**FIGURE 5:**
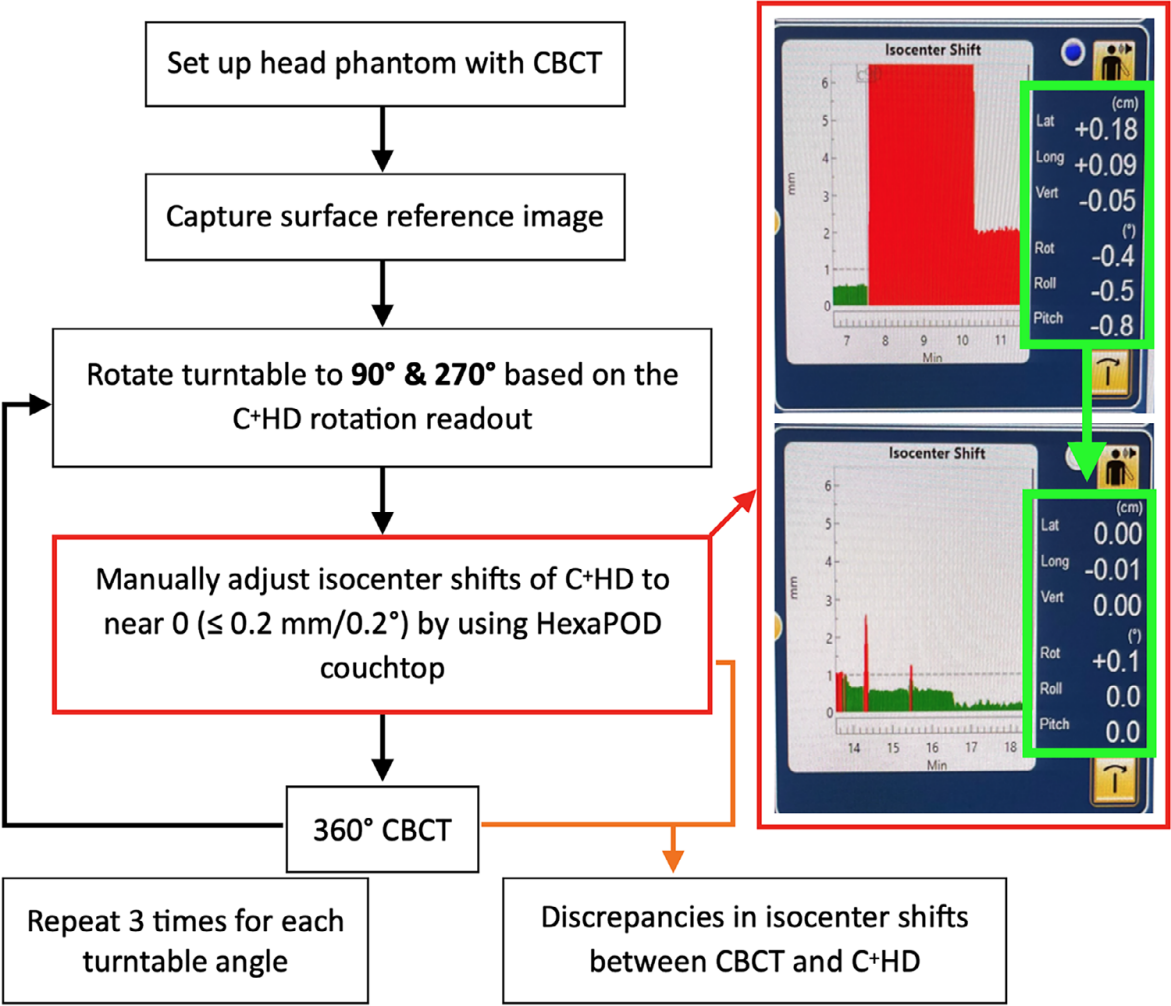
Flowchart of evaluating the accuracy of the FSRS system for correcting isocenter shifts. FSRS, frameless linear accelerator‐based stereotactic radiosurgery.

In the second method, we aimed to evaluate the accuracy of the FSRS system in correcting the isocenter shifts. During FSRS treatment, rotating the couch away from 0° may reduce the SG camera units' visibility of a portion of the ROI, especially at the couch angles of ± 90°.^14^ Therefore, the turntable was rotated to 90° (T90) and 270° (T270) (± 0.3°) based on the C^+^HD rotation readout. Subsequently, the isocenter shifts from the C^+^HD were manually adjusted to near zero in translational and rotational directions (≤ 0.2 mm/0.2°) using the HexaPOD couch control software. Following this adjustment, we performed CBCT and calculated the discrepancies in isocenter shifts between the CBCT and the C^+^HD. This procedure was repeated three times for both head phantoms. The workflow for this procedure is illustrated in Figure 5.

**FIGURE 6:**
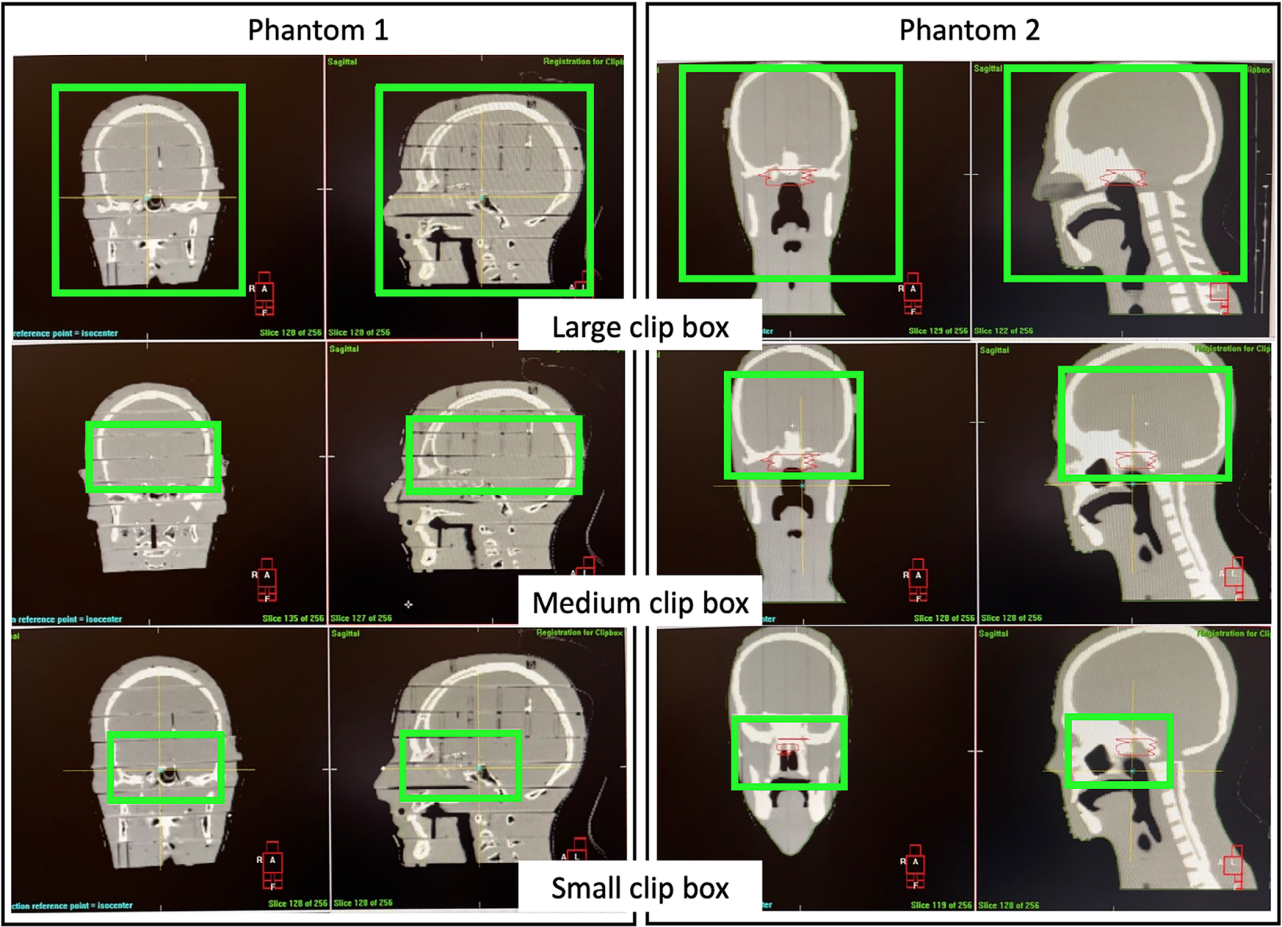
Different clip box volumes used for CBCT automatic registration on Phantom 1 and 2; the green square represents the clip box. CBCT, cone‐beam CT.

For the evaluations mentioned above, we used the clip box automatic registration with a predefined large clip box volume encompassing the entire head phantom. Theoretically, the larger clip box volume may yield more accurate registration results, but it also requires more time to process the CBCT‐CT registration. However, using different clip box volumes may introduce uncertainties in the image registration.^23^, ^24^ Consequently, we conducted further investigations to assess the effect of clip box volume on the accuracy of the FSRS system, using large, medium, and small clip box volumes at T90 and T270, as shown in Figure 6. These investigations followed the procedure for evaluating the ability of the FSRS system to correct isocenter shifts.

### 2.8 Error simulation

To evaluate the FSRS system's ability to detect position errors, we introduced errors by shifting the couch ± 2.0 mm in the three translational directions at seven different turntable angles (0°, 30°, 60°, 90°, 270°, 300°, and 330°). We then measured the isocenter shifts from the CBCT and the C^+^HD at these seven turntable angles. This procedure was repeated three times for both head phantoms.

## 3 RESULTS

Through the QA procedures mentioned above for system integration, we ensured that the isocenters of different systems, Versa HD linac (XVI CBCT & MV), HexaPOD, room laser, and C^+^HD, match within acceptable tolerance limits. The WL test results showed an average deviation of 0.6 mm (± 0.2 mm) for both the cone and the MLC‐shaped FS. For the HexaPOD couch level verification, the digital level showed 0°–0.05° readings in the roll and pitch directions when no phantom was on the HexaPOD couch. After the HexaPOD system alignment check with the MIMI phantom, the digital level showed an average of 0.32° (± 0.02°) in the roll direction. After aligning the PG phantom using CBCT, the digital level showed an average of 0.75° (± 0.08°) in the roll direction and 0.22° (± 0.06°) in the pitch direction.

### 3.1 CBCT‐CT image registration method

**FIGURE 7:**
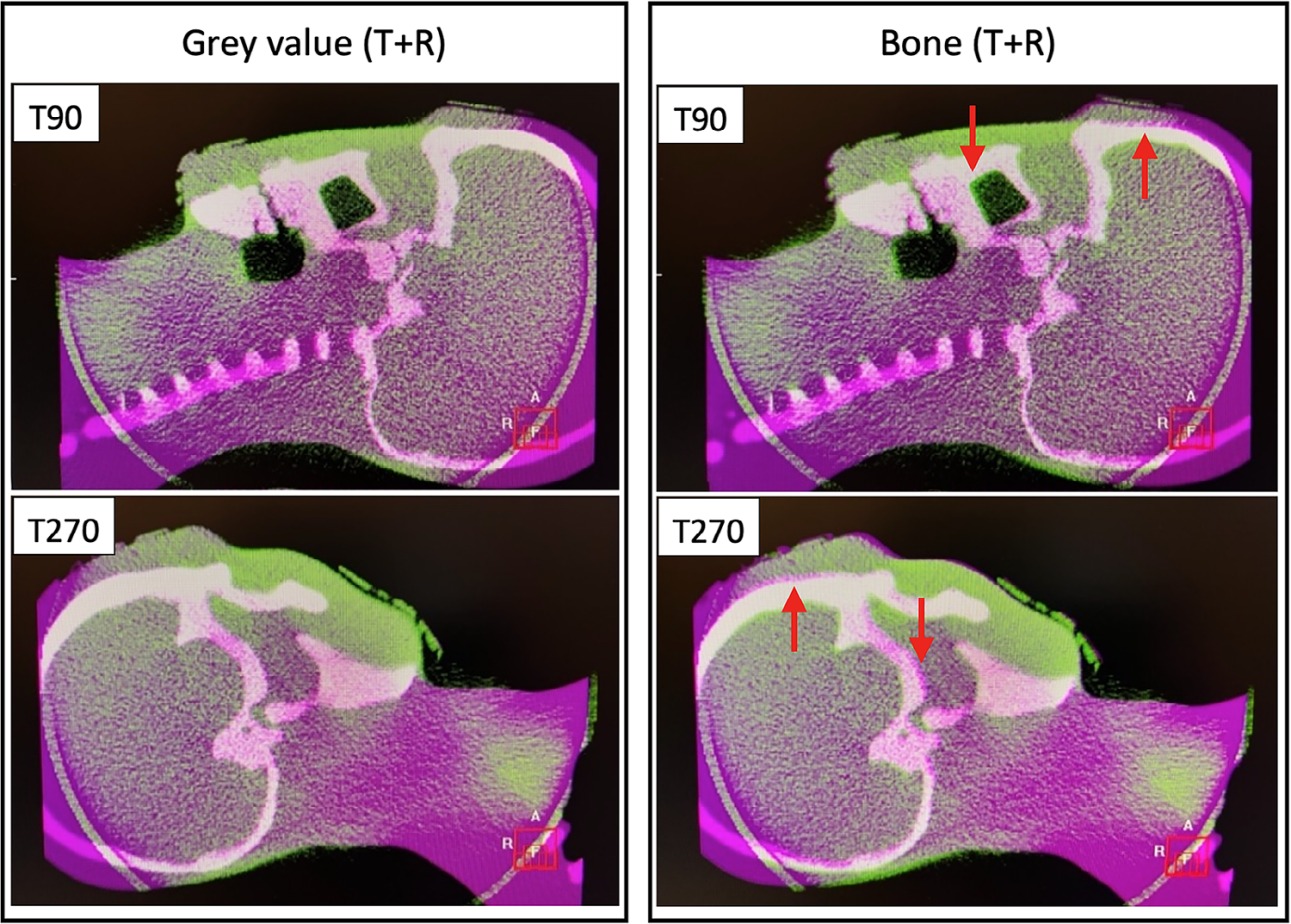
CBCT‐CT registration images with bone (T+R) mode registration method and gray value (T+R) mode registration method at turntable angles of 90° and 270°; the red arrows indicate the misalignments of bony structures. CBCT, cone‐beam CT.

Table 1 shows the average discrepancies between the two automatic registration methods in isocenter shifts. For Phantom1, the average discrepancies were all ≤ 0.2 mm/0.2° at different turntable angles. For Phantom 2, the average discrepancies were all ≤ 0.9 mm/0.5° at different turntable angles. More significant discrepancies were observed at T90 and T270 for Phantom 2 compared to Phantom 1. Therefore, we visually inspected the CBCT‐CT registration images after each registration method at these angles, and some misalignments of the bony structures were observed in the CBCT‐CT registration images with the BM registration method, as shown in Figure 7. Thus, we concluded that the GM registration method was more appropriate for evaluating the accuracy of the FSRS system with head phantoms. We used this registration method for the following measurements.

**TABLE 1:** Average discrepancies in isocenter shifts between (T+R) BM and the (T+R) GM registration methods at different turntable angles.

(BM‐GM)							
	T0	T30	T60	T90	T330	T300	T270
Phantom 1							
**(mm)**							
Lat.	0.1	0.1	0.0	0.1	0.0	0.0	0.0
Long.	0.2	0.0	0.0	0.0	0.0	0.0	0.0
Vert.	−0.1	−0.1	0.0	−0.1	0.0	0.0	0.0
**(°)**							
Pitch	0.0	0.1	0.0	−0.1	−0.1	0.0	−0.1
Roll	−0.1	−0.1	0.0	−0.1	−0.1	0.0	−0.1
Rot.	−0.1	−0.1	0.0	0.2	0.0	0.0	0.0
**Phantom 2**							
**(mm)**							
Lat.	−0.1	−0.1	0.0	−0.3	0.0	0.2	0.7
Long.	−0.1	−0.1	0.1	−0.2	0.1	0.2	0.2
Vert.	0.0	−0.1	0.1	0.9	−0.1	0.1	0.7
**(°)**							
Pitch	−0.1	−0.1	−0.1	−0.4	−0.1	−0.3	−0.5
Roll	−0.2	−0.1	−0.2	−0.1	−0.1	−0.3	−0.2
Rot.	0.1	0.0	0.1	−0.1	0.0	−0.2	−0.2

*Note*: Gray highlight indicates the largest discrepancy in translation and rotation for each phantom.

Abbreviations: BM, bone mode; GM, gray value mode; TX, turntable angle of X.

### 3.2 Accuracy of FSRS system based on two different routine QA procedures

Table 2 shows the average discrepancies in isocenter shifts between the CBCT and the C^+^HD using two different QA procedures at different turntable angles. The QA procedure of the PG phantom aligned to the room laser isocenter showed comparable results. Therefore, we used this procedure for the C^+^HD routine QA before evaluating the accuracy of the FSRS system with the head phantoms.

**TABLE 2:** Average discrepancies in isocenter shifts between CBCT and C^+^HD using two QA procedures at different turntable angles.

(CBCT‐C^+^HD)							
	T0	T30	T60	T90	T330	T300	T270
**PG phantom alignment using CBCT**							
**(mm)**							
Lat.	0.3	0.6	0.9	1.2	−0.2	−0.5	−0.4
Long.	0.1	−0.2	−0.6	−0.8	0.2	−0.5	−0.8
Vert.	−0.1	0.2	0.1	0.2	0.1	0.2	0.1
**(°)**							
Pitch	0.0	−0.1	−0.2	−0.2	−0.1	0.0	0.3
Roll	0.0	0.1	0.0	0.1	0.0	0.0	0.0
Rot.	0.2	0.2	−0.2	−0.3	0.0	−0.2	0.0
**PG phantom alignment using room lasers**							
**(mm)**							
Lat.	−0.2	0.1	−0.6	0.4	0.0	−0.1	0.3
Long.	−0.4	−0.3	0.9	−0.1	−0.1	−0.3	−0.5
Vert.	0.1	0.2	0.0	0.2	0.2	0.3	0.3
**(°)**							
Pitch	0.1	0.0	0.0	0.0	−0.3	−0.6	−0.5
Roll	0.1	0.0	−0.1	−0.5	0.1	0.2	0.0
Rot.	−0.1	0.0	−0.4	−0.4	−0.6	−0.5	−0.3

*Note*: Gray highlight indicates the discrepancy > 0.5 mm/0.5°.

Abbreviations: CBCT, cone‐beam CT; C^+^HD, Catalyst^+^ HD; TX, turntable angle of X; QA, quality assurance.

### 3.3 Accuracy of FSRS system

Table 3 shows the average discrepancies in isocenter shifts between the CBCT and the C^+^HD with two different ROI sizes for both head phantoms at different turntable angles. For both head phantoms with both ROI sizes, the average discrepancies were all < 1 mm/1°. The small ROI showed a trend toward more significant discrepancies in both translational and rotational directions with increased turntable angles for both phantoms. The large ROI showed fewer average discrepancies > 0.5 mm/0.5° for both phantoms.

**TABLE 3:** Average discrepancies in isocenter shifts between CBCT and C^+^HD with two different ROI sizes at different turntable angles.

Phantom 1, (CBCT‐C^+^HD)							Phantom 2, (CBCT‐C^+^HD)							
	T0	T30	T60	T90	T330	T300	T270	T0	T30	T60	T90	T330	T300	T270
**Small ROI size**														
**(mm)**														
Lat.	0.1	0.7	0.2	0.6	−0.1	0.0	0.2	−0.1	0.2	0.1	0.3	0.3	−0.2	−0.8
Long.	0.1	0.2	0.3	0.2	0.1	0.6	0.1	0.4	−0.2	−0.8	−0.7	0.4	0.9	0.8
Vert.	0.0	0.3	0.2	0.3	0.2	0.3	0.2	0.1	0.1	0.1	0.3	0.5	0.4	0.4
**(°)**														
Pitch	−0.1	−0.4	−0.6	−0.9	0.0	0.0	0.3	0.2	0.2	−0.2	−0.3	0.6	0.4	0.7
Roll	0.1	0.0	0.1	0.1	0.0	−0.6	−0.1	0.2	−0.1	−0.3	−0.2	−0.3	−0.2	0.0
Rot.	0.3	0.1	−0.5	−0.9	−0.6	−0.8	−0.8	−0.2	−0.2	−0.6	−0.7	−0.4	−0.1	−0.2
**Large ROI size**														
**(mm)**														
Lat.	−0.2	0.1	−0.6	0.4	0.0	−0.1	0.3	0.1	0.1	−0.1	−0.7	0.5	0.6	0.2
Long.	−0.4	−0.3	0.9	−0.1	−0.1	−0.3	−0.5	0.0	−0.3	−0.2	−0.1	0.0	0.3	0.5
Vert.	0.1	0.2	0.0	0.2	0.2	0.3	0.3	−0.4	−0.5	−0.3	−0.1	−0.1	−0.1	0.0
**(°)**														
Pitch	0.1	0.0	0.0	0.0	−0.3	−0.6	−0.5	0.0	−0.2	−0.4	−0.3	0.2	0.4	0.4
Roll	0.1	0.0	−0.1	−0.5	0.1	0.2	0.0	0.0	−0.1	0.0	−0.1	−0.2	−0.2	0.1
Rot.	−0.1	0.0	−0.4	−0.4	−0.6	−0.5	−0.3	0.1	0.0	0.0	0.0	0.1	0.1	0.0

*Note*: Gray highlight indicates the discrepancy > 0.5 mm/0.5°.

Abbreviations: CBCT, cone‐beam CT; C^+^HD, Catalyst^+^ HD; ROI, region of interest.

For evaluating the accuracy of the FSRS system in correcting isocenter shifts, Table 4 shows the average discrepancies in isocenter shifts between the CBCT and the C^+^HD with two different ROI sizes for both head phantoms at T90 and T270. For the small ROI size, the average discrepancies in rotational direction at T90 and T270 were ≥ 1° for Phantom 1. In contrast, the average discrepancies for the large ROI size were all < 1 mm/1° for both phantoms, with fewer discrepancies > 0.5 mm/0.5°. These results and the findings in Table 3 led to the decision to use the large ROI size for the subsequent measurements.

**TABLE 4:** Average discrepancies in isocenter shifts between CBCT and C^+^HD with two different ROI sizes at turntable angles of 90° and 270° for evaluating the accuracy of the FSRS system in correcting isocenter shift.

(CBCT‐C^±^HD)				
	Phantom 1		Phantom 2	
	T90	T270	T90	T270
**Small ROI size**				
**(mm)**				
Lat.	0.9	0.9	−0.3	0.9
Long.	0.8	0.0	−0.3	0.5
Vert.	0.3	0.2	0.4	0.2
**(°)**				
Pitch	−0.6	0.6	−0.3	0.2
Roll	0.1	0.1	−0.2	−0.2
Rot.	−1.3	−1.0	0.0	0.0
**Large ROI size**				
**(mm)**				
Lat.	0.4	0.3	−0.4	0.9
Long.	0.5	−0.1	0.3	0.2
Vert.	0.3	0.4	0.5	−0.1
**(°)**				
Pitch	−0.3	0.1	−0.1	0.4
Roll	0.2	0.4	0.0	0.1
Rot.	−0.8	−0.1	0.0	0.0

*Note*: Gray highlight indicates the discrepancy > 0.5 mm/0.5°.

Abbreviations: CBCT, cone‐beam CT; C^+^HD, Catalyst^+^ HD; ROI, region of interest.

Table 5 shows the average discrepancies in isocenter shifts between the CBCT with different clip box volumes for investigating the clip box volume effect. For Phantom 1, the average discrepancies between different clip box volumes were all ≤ 0.1 mm/0.1°. For Phantom 2, the average discrepancies between different clip box volumes were all ≤ 0.5 mm/0.7°, most of the average discrepancies between medium and large clip box volumes were ≤ 0.1 mm/0.1°.

**TABLE 5:** Average discrepancies in isocenter shifts between CBCT with different clip box volumes and C^+^HD with a large ROI size at turntable angles of 90° and 270°.

Phantom 1, (CBCT‐C^+^HD)							Phantom2, (CBCT‐C^+^HD)					
	(S‐M)		(S‐L)		(M‐L)		(S‐M)		(S‐L)		(M‐L)	
	T90	T270	T90	T270	T90	T270	T90	T270	T90	T270	T90	T270
**(mm)**												
Lat.	0.1	0.0	−0.1	0.0	−0.1	0.0	0.5	0.4	0.4	0.3	−0.1	0.3
Long.	0.0	−0.1	0.0	−0.1	0.0	0.0	−0.1	−0.1	−0.1	0.2	0.0	0.1
Vert.	0.1	0.0	−0.1	0.0	−0.1	0.0	0.0	0.1	0.1	0.5	0.1	−0.1
**(°)**												
Pitch	0.0	−0.1	0.0	−0.1	0.0	0.0	−0.7	−0.5	−0.6	−0.6	0.1	−0.1
Roll	0.0	0.0	0.1	0.0	0.1	0.0	0.0	−0.2	0.0	−0.3	0.0	−0.1
Rot.	0.0	−0.1	−0.1	−0.1	−0.1	0.0	−0.1	0.0	0.0	0.0	0.1	0.0

*Note*: Small/medium/large clip box volume; gray highlight indicates the discrepancy > 0.5 mm/0.5.

Abbreviations: CBCT, cone‐beam CT; C^+^HD, Catalyst^+^ HD; ROI, region of interest; S/M/L = small/medium/large.

### 3.4 Error simulation

Table 6 shows the average discrepancies in isocenter shifts between CBCT and C+HD when translational errors of ± 2.0 mm were introduced at different turntable angles. For Phantom 1, the average discrepancies were all ≤ 0.9 mm/0.3°. For Phantom 2, most of the average discrepancies were ≤ 0.9 mm/0.7°. However, at T270, the average translational discrepancies in the lateral direction were 1.1 mm and 1.3 mm for errors of +2 mm and −2 mm, respectively.

**TABLE 6:** Average discrepancies in isocenter shifts between CBCT and C^+^HD for errors of ± 2.0 mm at different turntable angles.

Phantom 1, (CBCT‐C^+^HD)								Phantom 2, (CBCT‐C^+^HD)						
	T0	T30	T60	T90	T330	T300	T270	T0	T30	T60	T90	T330	T300	T270
**2 mm**														
**(mm)**														
Lat.	0.2	0.5	0.2	0.8	0.0	−0.3	−0.2	−0.1	−0.3	−0.3	−0.2	0.4	0.7	1.1
Long.	0.5	0.1	−0.5	−0.1	0.7	0.7	0.1	−0.3	−0.2	0.0	0.1	−0.3	−0.1	0.1
Vert.	0.0	0.1	0.1	0.2	0.1	0.3	0.3	0.1	0.1	0.2	0.5	0.3	0.3	0.6
**(°)**														
Pitch	−0.1	0.0	0.0	−0.3	0.1	−0.1	0.1	0.1	0.0	−0.3	−0.3	0.3	0.4	0.7
Roll	0.1	−0.1	−0.2	−0.1	−0.2	0.0	0.3	0.2	0.3	0.2	0.3	0.1	−0.1	0.2
Rot.	0.3	0.3	0.0	0.1	−0.3	0.1	0.1	−0.1	0.3	0.0	−0.1	0.2	0.2	0.2
**−2 mm**														
**(mm)**														
Lat.	0.2	0.5	0.5	0.6	−0.2	−0.4	0.0	0.0	−0.2	−0.2	−0.1	0.2	0.9	1.3
Long.	0.6	−0.1	−0.5	−0.1	0.9	0.6	0.1	−0.4	−0.1	0.0	0.2	−0.3	−0.2	0.2
Vert.	0.0	0.0	0.1	0.1	0.1	0.3	0.1	0.0	−0.1	0.1	0.2	0.2	0.3	0.4
**(°)**														
Pitch	0.0	0.0	0.0	−0.3	−0.1	−0.1	−0.1	0.1	0.0	−0.2	−0.2	0.2	0.4	0.7
Roll	0.0	0.1	−0.1	−0.1	0.0	0.0	0.2	0.2	0.3	0.3	0.3	0.1	0.0	0.2
Rot.	0.1	0.1	0.2	−0.1	0.0	0.2	0.1	0.0	0.1	−0.1	−0.1	0.3	0.3	0.1

*Note*: Gray highlight indicates the discrepancy > 0.5 mm/0.5°.

Abbreviations: CBCT, cone‐beam CT; C^+^HD, Catalyst^+^ HD.

## 4 DISCUSSION

We presented our integrated system for FSRS treatment: Versa HD linac (XVI CBCT and MV beam), HexaPOD, room laser, and C^+^HD. Through our system QA procedures, we ensured that the isocenters of these systems match within tolerance limits. These procedures can be considered a reference for establishing the commissioning process for the FSRS system.

After performing the MV‐kV coincidence check, the CBCT isocenter can be regarded as the standard for verifying isocenters of the other systems. However, the XVI CBCT has some image registration uncertainties from the automatic image registration method and the clip box volume. In addition, it has been demonstrated that the XVI CBCT exhibits a rotational artifact attributed to the mislabeled projection data. This artifact results in a slight rotation of the CBCT image in the opposite direction of the scan, approximately 0.25° in the roll direction per 360° scan.^26^ As a result, aligning the phantom's CBCT image with the reference CBCT image would cause a slight rotation of the HexaPOD couch. Therefore, we used a digital level to verify the level of the HexaPOD couch after applying the HexaPOD couch shifts. The digital level showed an average of 0.32° in the roll direction after the HexaPOD system alignment check with the MIMI phantom, which was consistent with the findings of Ali et al.^26^

A solution to the XVI CBCT's rotational artifact involves the implementation of a manufacturer‐provided software correction, contingent upon its availability. Other clinical solutions include increasing the scan time, narrowing the x‐ray pulse width while maintaining mAs, or applying a single angle calibration value to all CBCT scans. However, each solution has drawbacks, so meticulous evaluations are necessary before implementation.^26^ We believe that a calibrated digital level is essential for quantifying the XVI CBCT's rotational artifact and other rotational uncertainties resulting from applying 6‐DoF couch corrections.

For the C^+^HD routine QA, the PG phantom is typically used to ensure the alignment between the C^+^HD isocenter and the CBCT isocenter. However, if users cannot obtain the reference CT images of the PG phantom from the vendor for CBCT‐CT image registration, there may be some uncertainties when scanning the phantom on their own, such as CT couch level, phantom alignment, slice thickness, and so forth. We also used the digital level to measure the HexaPOD couch's level after aligning the PG phantom using CBCT. These measurements showed an average of 0.75° in the roll direction and 0.22° in the pitch direction. This indicates other uncertainties in the CBCT‐CT image registration for the PG phantom besides the rotational artifact, which could be caused by the PG phantom CT scan accuracy in the institution. In this study, we compared two different routine QA procedures: one using CBCT and the other using room lasers for the PG phantom alignment. Our results have demonstrated that it is feasible to use room lasers to ensure the alignment of the C^+^HD isocenter with the CBCT isocenter. In addition, in clinical practice, once the patient is aligned based on the CBCT‐CT registration images, a surface image of the patient is captured. Although the routine QA process corrects the system difference between C^+^HD and CBCT, this correction becomes irrelevant after capturing the patient's surface image. Therefore, we believe users can use room lasers in C^+^HD routine QA. This approach helps users save time by not performing CBCT scans of the PG phantom.

During noncoplanar FSRS, using CBCT to verify patient positioning at different couch angles has limitations. Alternatively, stereoscopic x‐ray imaging or SG systems can check patient positioning accuracy at different couch angles during treatment. It is essential to perform QA procedures to ensure the accuracy of these systems.

For noncoplanar SIMT FSRS, the SG system has demonstrated its ability to improve treatment accuracy, as evidenced by the patient‐specific QA results.^25^ However, to our knowledge, no study has quantified the accuracy of the FSRS system based on the isocenter shifts from the CBCT at different couch angles. Therefore, in this study, we overcame the limitation of performing a 360°CBCT scan at these rotation angles by using a turntable to simulate couch rotation.

The accuracy of the C^+^HD or any image‐guided system is difficult to measure independently because, in this study, the phantom was placed on the HexaPOD couch, set up using CBCT, and then the surface image of the phantom was captured using the C^+^HD. Therefore, acquiring the isocenter shifts from the C^+^HD involves multiple systems, and each system needs to be integrated through a series of QA procedures. In this study, we have emphasized that our FSRS system incorporates different systems and the associated integration process. Thus, we evaluated the accuracy of the FSRS system by comparing the isocenter shifts from the two systems.

We evaluated the accuracy of the FSRS system using two methods. Both methods involved the following steps: rotating the turntable, performing a 360°CBCT scan, and calculating the discrepancies in isocenter shifts between the CBCT and the C^+^HD. The main distinctions occurred in the second method, where we zeroed out the isocenter shifts from the C^+^HD before performing the CBCT scans, and we focused on only two large turntable angles (T90 and T270). This allowed us to evaluate the accuracy of the FSRS system in correcting the isocenter shifts at T90 and T270. However, some uncertainties may affect the accuracy of the FSRS system, including the CBCT registration method, CBCT clip box volume, and selected ROI size in the C^+^HD system.

For the CBCT‐CT image registration method, the BM registration method can be slightly faster than the GM registration method due to its insensitivity to image noise.^21^ However, Meyer et al. demonstrated that, for the head phantom, the GM registration method was more accurate than the BM registration method.^27^ Our results also showed that the GM registration method performed better, and we used the GM registration method to evaluate the accuracy of the FSRS system.

Our results for the clip box automatic registration with the GM registration method indicated that the variations between different clip box volumes were small for Phantom 1. However, for Phantom 2, the variations between the small clip box volume and the other two clip box volumes were slightly larger. We speculated that it might be due to the phantom's homogeneity. Phantom 1 exhibited more inhomogeneity than Phantom 2 (Figure 6), implying that more image pixel density variations were involved during the image registration. This could lead to more accurate registration results because the registration algorithm has more information to use for better alignment. Based on our results, a large clip box volume for FSRS treatment is recommended.

As the clinical practice standard for patient positioning, XVI CBCT has uncertainties, including those associated with the registration method and clip box volume mentioned above. Therefore, we attempted to minimize these uncertainties before evaluating the accuracy of the FSRS system. Despite these efforts, the rotational artifact of XVI CBCT remained in this study.

For the ROI size in the C^+^HD system, our results indicated that the average discrepancies in isocenter shifts between the CBCT and the C^+^HD were all < 1 mm/1° for the large ROI size, and the large ROI size also showed fewer discrepancies > 0.5 mm/0.5°. During the measurements, we observed that a small ROI size can lead to significant fluctuations in the C^+^HD readouts, which may affect the accuracy of the FSRS system. The SGRT system may also generate inaccurate patient position corrections in response to facial movements, especially when using smaller ROIs.^28^ Despite a larger ROI resulting in a slower monitoring rate,^14^ we still recommend using a larger ROI size for FSRS treatment due to the high demand for accuracy and precision. The findings regarding uncertainties in each system from this study underscore the importance of establishing QA procedures for the FSRS system to ensure its accuracy.

To determine whether the C^+^HD‐based FSRS system has the potential to ensure effective FSRS treatment, further investigation into the overall FSRS uncertainties is needed. After aligning the patient at the couch angle of 0° during treatment, the uncertainty in couch rotation treatment arises from the couch's mechanical movement when applying the couch shifts at different couch angles. Therefore, we do not need to consider uncertainties from the room laser isocenter alignment, the HexaPOD isocenter alignment, and the C^+^HD routine QA during treatment. The overall uncertainties account for those associated with the MV‐kV coincidence, the couch movement accuracy, the CBCT‐CT image registration accuracy, and the accuracy of the FSRS system.

Based on our QA experience, after performing the CBCT scan of the phantom and applying the isocenter shifts using the HexaPOD couch, the isocenter shifts from the subsequent CBCT scan are always within 0.1 mm/0.1°. This indicates that the HexaPOD couch's movement is accurate. The image guidance (IG) uncertainties during FSRS treatment combine the CBCT‐CT image registration and the C^+^HD system. If needed, the XVI CBCT rotational artifact can be manually adjusted in clinical procedures. A previous study showed that the CBCT‐CT image registration errors for the head phantom were below 0.2 mm/0.2° with the XVI system.^27^ Since there are more image pixel density variations in real patients’ head images than in the head phantoms’ images, we expect more accurate alignment results from the CBCT‐CT image registration for real patients.

For the accuracy of the FSRS system, we demonstrated that by incorporating C^+^HD with the appropriate CBCT‐CT image registration method and ROI size of C^+^HD, the discrepancies in the isocenter shifts between the CBCT and the C^+^HD were ≤ 0.9 mm/0.8° at different turntable angles in this study. In addition, our QA results for the MV‐kV coincidence usually show values of ≤ 0.5 mm and the WL test results showed an average deviation of 0.6 mm for both the cone and the MLC‐shaped FS. Therefore, the overall accuracy of the FSRS system was on the order of 1.1 mm/1°.

Previous studies have indicated that, for linac‐based SRS with an immobilization mask, the intra‐fractional motion was ≤ 1 mm/1°.^27^, ^30^, ^31^, ^32^ Therefore, for the error simulation, we introduced errors of ± 2.0 mm in translational directions at different turntable angles to conservatively evaluate the ability of the FSRS system to detect position errors. Our results showed that the FSRS system, with the appropriate CBCT‐CT image registration method and ROI size of C^+^HD, accurately detected the position errors at different turntable angles, most of the average discrepancies in isocenter shifts were ≤ 0.9 mm/0.7°. The largest discrepancy in the isocenter shifts was up to 1.3 mm, which could result in the overall accuracy of the FSRS system exceeding 1 mm. However, we anticipate that in clinical practice, the overall accuracy of the FSRS system should remain on the order of 1.1 mm/1° due to the smaller patient position errors.

The TG‐302 report suggests adopting tighter patient positioning tolerances (≤ 1 mm/0.5°) for SIMT FSRS treatment.^14^ However, achieving the 0.5° tolerance limit is challenging in clinical practice with the SG‐based FSRS system, given our above results. Therefore, further evaluations are warranted.

## 5 CONCLUSION

This study introduces an innovative approach to quantifying the impact of couch rotation on the accuracy of the FSRS system using head phantoms with turntables. Our results demonstrate that the SG‐based FSRS system can provide accurate isocenter shifts and detect translational and rotational errors at different turntable angles. The overall accuracy of the FSRS system was on the order of 1.1 mm/1°.

## AUTHOR CONTRIBUTIONS

Hao‐Wen Cheng and Chihray Liu conceived and designed the study. Hao‐Wen Cheng conducted the measurements and performed the data analysis with assistance from Sheng‐Hsuan Sun and Chihray Liu. Hao‐Wen Cheng wrote the paper with assistance from Jonathan Li and Guanghua Yan.

## CONFLICT OF INTEREST STATEMENT

The authors declare that they have no conflicts of interest.
